# Theoretical insights into the thermally activated delayed fluorescence of pyridazine derivatives: balancing twist and charge-transfer character

**DOI:** 10.1039/d6ra07215a

**Published:** 2026-10-08

**Authors:** Yan Liu, Shuhua Ma, Yuanyuan Yang, Chunhua Qu, Junyan Zhou, Yushan Wang, Yibin Yang, Weiguo Cao

**Affiliations:** a School of Chinese Materia Medica, Chongqing College of Traditional Chinese Medicine Chongqing 402760 China caoweiguo@cqctcm.edu.cn; b Chongqing Traditional Chinese Medicine Hospital Chongqing 400021 China; c Chemical Pollution Control Chongqing Applied Technology Extension Center of Higher Vocational Colleges, Chongqing Industry Polytechnic University Chongqing 401120 China yangyb@cqipu.edu.cn; d Modern Chinese Medicine Industry College, Chongqing College of Traditional Chinese Medicine Chongqing 402760 China

## Abstract

In this work, a systematic theoretical study of 4,5,6-triphenylpyridazine-3-carbonitrile-based (TPPz-based) donor–acceptor molecules reveals that donor steric bulk critically controls molecular planarity and excited-state properties. The moderately bulky electron-donating 4-(bis(4-(*tert*-butyl)phenyl)amino)phenyl (BPA) moiety induces the largest overall molecular distortion and yields a nearly pure CT S_1_ state together with an HLCT T_1_ state. In contrast, the rigid pyrenyl donor in 4,6-diphenyl-5-(4-(pyren-1-yl)phenyl)pyridazine-3-carbonitrile (Pyr-TPPz) gives a CT S_1_ state but a locally excited T_1_ state. 5-(4-(bis(4-(*tert*-butyl)phenyl)amino)phenyl)-4,6-diphenylpyridazine-3-carbonitrile (BPA-TPPz) exhibits the lowest reorganization energy (2513 cm^−1^), efficiently suppressing nonradiative decay, and shows the highest *k*_risc_ among the series. TPPz and Pyd-TPPz exhibit moderate RISC, whereas Pyr-TPPz shows a very low *k*_RISC within the S_1_–T_1_ two-state approximation because of its large S_1_–T_1_ gap, despite its LE T_1_ character. These results establish that optimal thermally activated delayed fluorescence performance requires a delicate balance between a twisted architecture for exciton management, suitable donor–acceptor electronic structures to modulate charge-transfer/locally excited (CT/LE) hybridization, and flexible donors to minimize reorganization energy. These findings provide clear structure–property guidelines for the design of efficient thermally activated delayed fluorescence (TADF) materials.

## Abstract 2

In this work, a systematic theoretical study of 4,5,6-triphenylpyridazine-3-carbonitrile-based (TPPz-based) donor–acceptor molecules reveals that donor steric bulk critically controls molecular planarity and excited-state properties.

## Graphical abstract

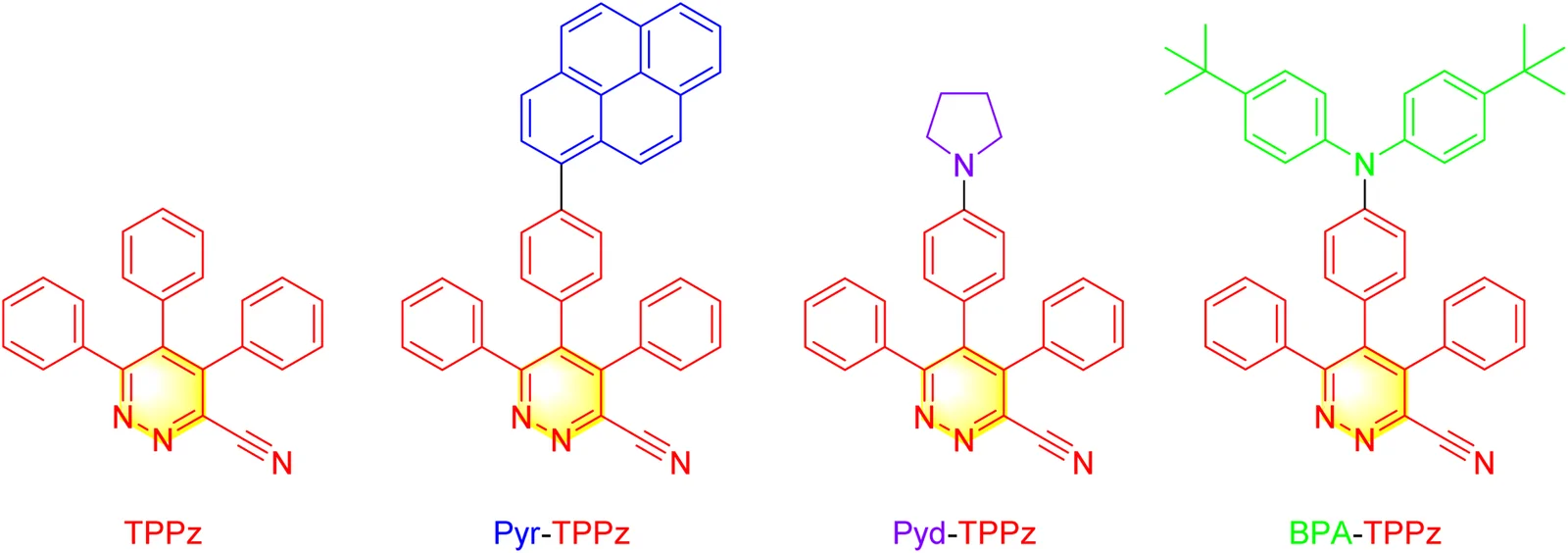

## Introduction

Organic light-emitting diodes (OLEDs) have emerged as a mainstream light source in the flat-panel display industry, owing to the tunability of their luminescent properties—such as color and operational lifetime—through rational molecular design.^1–5^ Nevertheless, raising luminescence efficiency remains the central challenge in the development of OLED emitters. First-generation fluorescent materials are limited to a maximum theoretical internal quantum efficiency of 25%, while second-generation phosphorescent emitters can, in principle, reach 100%. Thermally activated delayed fluorescence (TADF) materials, now recognized as the third generation of OLED luminophores,^6–13^ can likewise realize a theoretical quantum yield of 100% by harvesting both singlet and triplet excitons. Crucially, unlike phosphorescent complexes, TADF emitters can be constructed entirely from pure organic molecules, eliminating the need for costly noble metals such as platinum and iridium and thereby reducing production costs.^5^ Furthermore, organic scaffolds are readily modified, and their luminescence characteristics can be systematically tailored by introducing different functional groups.

The operational principle of TADF relies on the efficient up-conversion of non-emissive triplet excitons to emissive singlet states *via* reverse intersystem crossing (RISC).^11,12^ In a three-state model, a small energy gap Δ*E*_ST_ between the lowest excited singlet (S_1_) and triplet (T_1_) states is a prerequisite for efficient RISC. The most common design strategy to minimize Δ*E*_ST_ is the introduction of electron-donor (D) and electron-acceptor (A) moieties, which substantially reduces the spatial overlap between the highest occupied molecular orbital (HOMO) and the lowest unoccupied molecular orbital (LUMO).^14,15^ Pure organic molecules typically exhibit weak spin–orbit coupling (SOC) and thus sluggish intersystem crossing (ISC); SOC can be enhanced by incorporating heteroatoms (*e.g.*, N, S, P) or functional groups (*e.g.*, carbonyl, sulfonyl), and by exploiting the El-Sayed rule through the mixing of states with different electronic character.^16–18^ A landmark achievement was reported in 2012 by Adachi and co-workers, who realized a series of purely organic TADF emitters with near-unity photoluminescence quantum yields,^2^ igniting intense research on high-performance TADF materials. Subsequently, Zhang *et al.* developed two coumarin-based TADF molecules with exceptionally small singlet–triplet splittings, achieving external quantum efficiencies of 17.8% and 19.6%, respectively.^19^ To further boost device performance, diverse molecular design strategies have been pursued,^20,21^ as exemplified by the organoboron blue emitters reported by Kondo *et al.*,^21^ in which the HOMO is localized on nitrogen atoms and the *meta* positions of boron, while the LUMO resides at the *ortho* and *para* positions, thereby reducing the HOMO–LUMO spatial overlap.^22–27^

According to Fermi's golden rule, the RISC rate is inversely proportional to Δ*E*_ST_ and scales with the SOC strength. The prevailing D–A architecture exploits steric hindrance at the D–A junction to induce a twisted conformation that spatially separates the HOMO and LUMO and thus minimizes Δ*E*_ST_. Excessive twisting, however, can reduce the radiative transition rate and aggravate nonradiative decay. Moreover, although a twisted scaffold effectively suppresses exciton annihilation caused by intermolecular π–π stacking, it may simultaneously amplify excited-state geometric relaxation. The central challenge in realizing highly efficient TADF therefore lies in precisely balancing molecular distortion and donor–acceptor electronic coupling.^28–31^ TPPz, with its multiple rotatable benzene rings and tunable electron affinity, serves as an ideal acceptor platform for constructing D–A-type TADF emitters. By introducing donors with distinct steric demands and electronic characters, one can systematically modulate molecular conformation, charge-transfer (CT) character, and excited-state dynamics. Yet, a clear theoretical picture of how the rigidity/flexibility and electron-withdrawing/donating nature of the donors cooperatively govern molecular planarity, excited-state decay pathways, and ultimate TADF performance has remained elusive.

To address this gap, we herein employ 4,5,6-triphenylpyridazine-3-carbonitrile (TPPz)as the acceptor and select rigid pyrenyl (Pyr) donor, rigid electron-donating 4-(pyrrolidin-1-yl)phenyl (Pyd), and flexible electron-donating 4-(bis(4-(*tert*-butyl)phenyl)amino)phenyl (BPA) as donors, yielding three donor–acceptor systems (Pyr-TPPz, Pyd-TPPz, and BPA-TPPz) together with the parent acceptor TPPz as a reference. The ground state (S_0_), the first singlet excited state (S_1_), and the first triplet excited state (T_1_) were optimized using density functional theory (DFT) and time-dependent DFT (TD-DFT). Molecular planarity was quantified through the molecular planarity parameter (MPP) and the span of deviation from the plane (SDP), while root-mean-square displacement (RMSD) values were employed to evaluate excited-state geometric relaxation—all computed with the Multiwfn program. Electrostatic potential mapping and natural transition orbital (NTO) analysis, together with the locally excited (LE) proportion criterion, were performed to assign the excited-state character as CT, hybrid local and charge-transfer (HLCT), or LE. SOC constants between S_1_ and T_1_ were calculated using the quadratic response function method in the Dalton package, and the ISC and RISC rates were derived from time-dependent vibrational coupling (TVCF) theory. Furthermore, Huang–Rhys factors, reorganization energies, and Duschinsky rotation matrices were analyzed to elucidate the role of vibrational mode coupling in nonradiative decay and fluorescence quenching. Our results reveal that donor steric hindrance dictates the molecular conformation: Pyr-TPPz and BPA-TPPz adopt highly twisted geometries that efficiently suppress deleterious intermolecular π–π stacking, whereas TPPz and Pyd-TPPz retain good planarity. Crucially, the flexible electron-donating BPA moiety achieves the most pronounced geometric twisting and the highest degree of charge-transfer character in S_1_ (nearly pure CT), while the rigid pyrenyl (Pyr) donor also yields a CT S_1_ state but with a locally excited T_1_ state. Although BPA-TPPz undergoes the largest ground-to-excited-state geometric relaxation, its reorganization energy is substantially reduced owing to a redistribution into low-frequency vibrations enabled by the flexibility of BPA, thereby effectively suppressing nonradiative decay. Among the series, BPA-TPPz exhibits the highest *k*_risc_, followed by TPPz and Pyd-TPPz, whereas Pyr-TPPz exhibits a very low *k*_risc_ within the S_1_–T_1_ two-state approximation because its large S_1_–T_1_ gap outweighs the SOC enhancement from its LE T_1_ character. These trends are in excellent agreement with experimental observations. These findings clearly elucidate how donor steric and electronic effects synergistically regulate excited-state dynamics and provide a theoretical foundation for the fine balancing of twisted architecture, CT character, and vibrational coupling through molecular engineering toward high-performance TADF materials.

## Computational details

Ground-state (S_0_) and excited-state (S_1_ and T_1_) geometries of TPPz, Pyr-TPPz, Pyd-TPPz, and BPA-TPPz were optimized using DFT and TD-DFT, respectively.^32–35^ Frequency calculations were performed for the optimized S_0_, S_1_, and T_1_ structures to confirm that they correspond to minima; no imaginary frequencies were found. It is well recognized that the accurate prediction of the energy gap (Δ*E*_ST_) and excited-state properties of TADF molecules remains challenging. Because TADF molecules often possess significant CT character, the choice of functional for excited-state calculations is particularly important. Previous benchmark studies have shown that range-separated functionals such as CAM-B3LYP provide a more balanced description of CT excitations than global hybrids such as B3LYP and PBE0.^31,32^ In this work, CAM-B3LYP/def2-SVP was therefore used for geometry optimization and qualitative CT/HLCT assignment. Literature benchmarks for TADF-related CT states typically show deviations of about 0.1–0.3 eV;^31,33,34^ therefore, the absolute excited-state energies reported here should be regarded as qualitative and require validation by higher-level methods for quantitative purposes. Considering the balance between computational cost and the need for a balanced description of CT states, CAM-B3LYP was selected to describe the ground- and excited-state properties of TPPz, Pyr-TPPz, Pyd-TPPz, and BPA-TPPz. All calculations were performed using Gaussian 16.^36^ The hole–electron isosurfaces, heat maps, and natural transition orbitals (NTOs) were analyzed using Multiwfn.^37–39^

The Visual Molecular Dynamics (VMD) program is used for graphical and visual analysis.^40^ Based on the above data, the MOMAP program was used to calculate the Huang–Rhys (HR) factor, reorganization energy, the radiative rate, the non-radiative decay rate and ISC rate,^41–43^ while the Dalton package was used to calculate the SOC.^44,45^

## Results and discussions

The optimized geometrical structures of TPPz, Pyr-TPPz, Pyd-TPPz, and BPA-TPPz are presented in Fig. 1. As shown, each TPPz acceptor bears three rotatable benzene units linked to the pyridazine core, and two additional rotational dihedral angles exist at the junctions between the donors (Pyr, Pyd, and BPA) and the acceptor (TPPz). To quantify the twist, we measured the key dihedral angle *θ*_D–A_ between the donor unit and the adjacent TPPz phenyl ring, as defined in Fig. 1. The *θ*_D–A_ values for the four molecules in their S_0_, S_1_, and T_1_ states are summarized in Table S1. In the S_0_ state, BPA-TPPz exhibits the largest *θ*_D–A_ (57.6°), followed by Pyd-TPPz (56.2°), Pyr-TPPz (53.6°), and TPPz (51.0°). In the S_1_ state, Pyr-TPPz shows the largest *θ*_D–A_ (59.5°), followed by BPA-TPPz (59.1°), Pyd-TPPz (56.5°), and TPPz (53.8°). In the T_1_ state, Pyd-TPPz possesses the largest *θ*_D–A_ (57.3°), followed by BPA-TPPz (56.4°), Pyr-TPPz (54.0°), and TPPz (51.2°). Overall, the *θ*_D–A_ values range from 51.0° to 59.5°, indicating moderate twisting across the series. The *θ*_D–A_ values are comparable among BPA-TPPz, Pyr-TPPz, and Pyd-TPPz, while TPPz consistently shows the smallest *θ*_D–A_ These dihedral angles render the entire molecules twisted to different degrees and give rise to differing molecular planarity, which in turn modulate the HOMO–LUMO spatial separation and CT character.

**Fig. 1:**
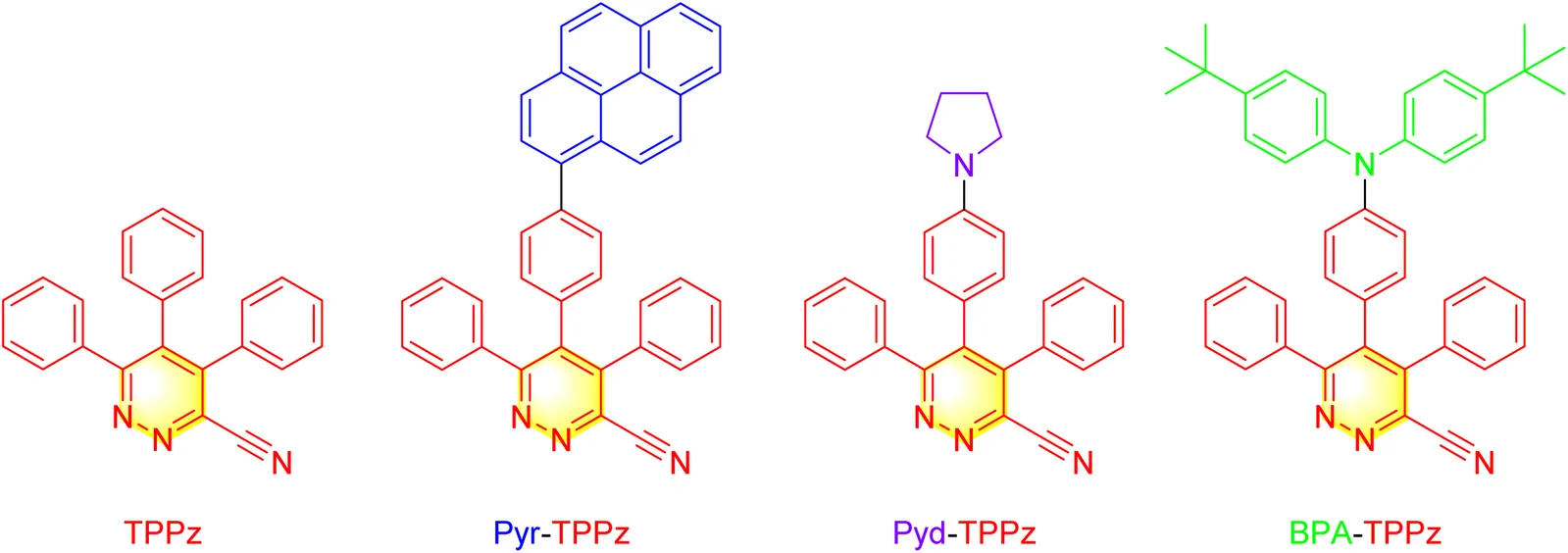
Chemical structure of TPPz, Pyr-TPPz, Pyd-TPPz and BPA-TPPz molecule, respectively.

To further evaluate the rigidity/flexibility of the molecular frameworks, we examined the variation of *θ*_D–A_ among the S_0_, S_1_, and T_1_ states. The changes from S_0_ to S_1_ are +2.8°, +5.9°, +0.3°, and +1.5° for TPPz, Pyr-TPPz, Pyd-TPPz, and BPA-TPPz, respectively, while the changes from S_0_ to T_1_ are +0.2°, +0.4°, +1.1°, and −1.2°. The differences between the S_1_ and T_1_ states are 2.6°, 5.5°, 0.8°, and 2.7° for the four molecules. All these variations are smaller than 6°, indicating that none of the molecules undergoes significant torsional relaxation upon excitation. Therefore, all four molecules possess relatively rigid frameworks. Among them, Pyr-TPPz exhibits the largest variation in the local donor–acceptor dihedral angle, indicating the highest torsional flexibility at the D–A junction, whereas BPA-TPPz shows the largest overall geometric relaxation (RMSD). Such rigidity is beneficial for suppressing non-radiative decay caused by excited-state torsional motion and for maintaining good geometrical matching between the S_1_ and T_1_ states, which favors reverse intersystem crossing (RISC). The relatively larger S_1_/T_1_ dihedral mismatch in Pyr-TPPz may increase the structural reorganization energy and thus adversely affect RISC. These structural insights help to establish the relationship between molecular twisting, framework rigidity, and the observed TADF-related properties.

To further analyze this issue, the molecular planarity parameter (MPP) and the span of deviation from the plane (SDP) for all non-hydrogen atoms were evaluated in toluene using Multiwfn; the corresponding results are collected in Table S2. The MPP is defined as 
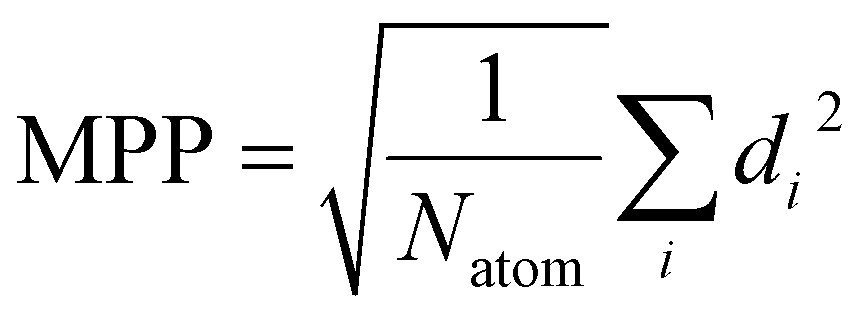
, where *d*_*i*_ is the distance of the *i*-th atom from the best-fit plane, providing a measure of the overall planarity of the region considered. The SDP is defined as SDP = *d*_max_ − *d*_min_, representing the maximum span perpendicular to the fitting plane. The MPP and SDP values for TPPz (0.67 Å and 2.26 Å) and Pyd-TPPz (0.66 Å and 2.20 Å) are the smallest among the series, whereas Pyr-TPPz (1.13 Å and 4.90 Å) and BPA-TPPz (2.15 Å and 9.98 Å) exhibit markedly larger values. In particular, BPA-TPPz is the only system for which both MPP and SDP exceed 1 Å and 6 Å, respectively; Pyr-TPPz has MPP > 1 Å but SDP < 6 Å. The considerable steric bulk of Pyr and BPA imposes severe steric hindrance in Pyr-TPPz and BPA-TPPz, forcing the Pyr and BPA moieties to adopt abnormally large dihedral angles with respect to the TPPz plane. This in turn leads to excessively large MPP and SDP values. Hence, high torsion with significant distortion relative to the plane fitted for the whole molecule is observed, highlighting the crucial role of the acceptor units in molecular twisting. Consequently, TPPz and Pyd-TPPz exhibit better molecular planarity. In addition, the highly twisted molecular configurations of all studied systems can weaken intermolecular π–π interactions and thereby suppress exciton annihilation—a necessary prerequisite for enabling TADF properties, as corroborated by additional evidence provided in the following sections.

We first optimized the geometries of TPPz in the ground state (S_0_), the first singlet excited state (S_1_), and the first triplet excited state (T_1_). The geometric changes between these states were quantified by the root-mean-square displacement (RMSD), calculated with Multiwfn as 
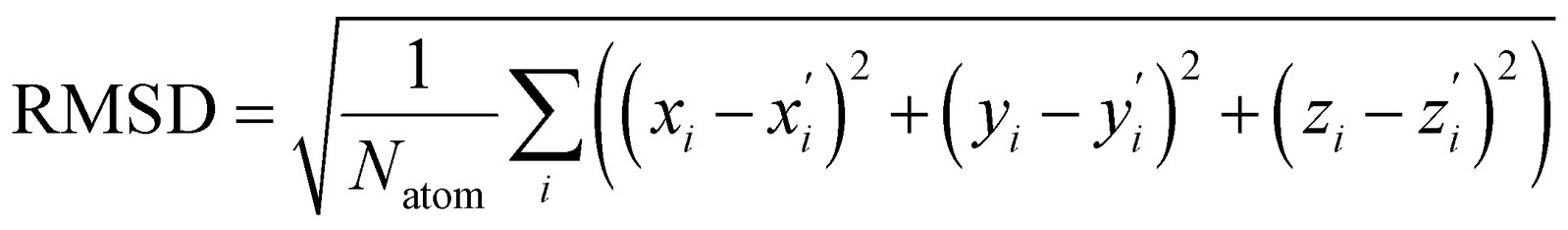
. The RMSD between S_0_ and S_1_ for BPA-TPPz is as large as 1.137 Å, far exceeding those of the other three compounds (0.152–0.181 Å). Conventionally, large structural distortion upon excitation is expected to accelerate internal conversion. However, as shown in Table S3, BPA-TPPz possesses the lowest internal conversion rate (*k*_ic_ = 1.5 × 10^10^ s^−1^), nearly 40 times smaller than that of TPPz. This apparent paradox is resolved by considering the reorganization energy: although BPA-TPPz undergoes significant geometric relaxation, the flexible BPA moiety redistributes the reorganization energy into low-frequency modes, drastically reducing the overall vibronic coupling and thus suppressing nonradiative decay. This demonstrates that the magnitude of geometric distortion alone is an unreliable predictor of nonradiative decay; rather, the distribution of reorganization energy among vibrational modes is the decisive factor (Fig. 2).

**Fig. 2:**
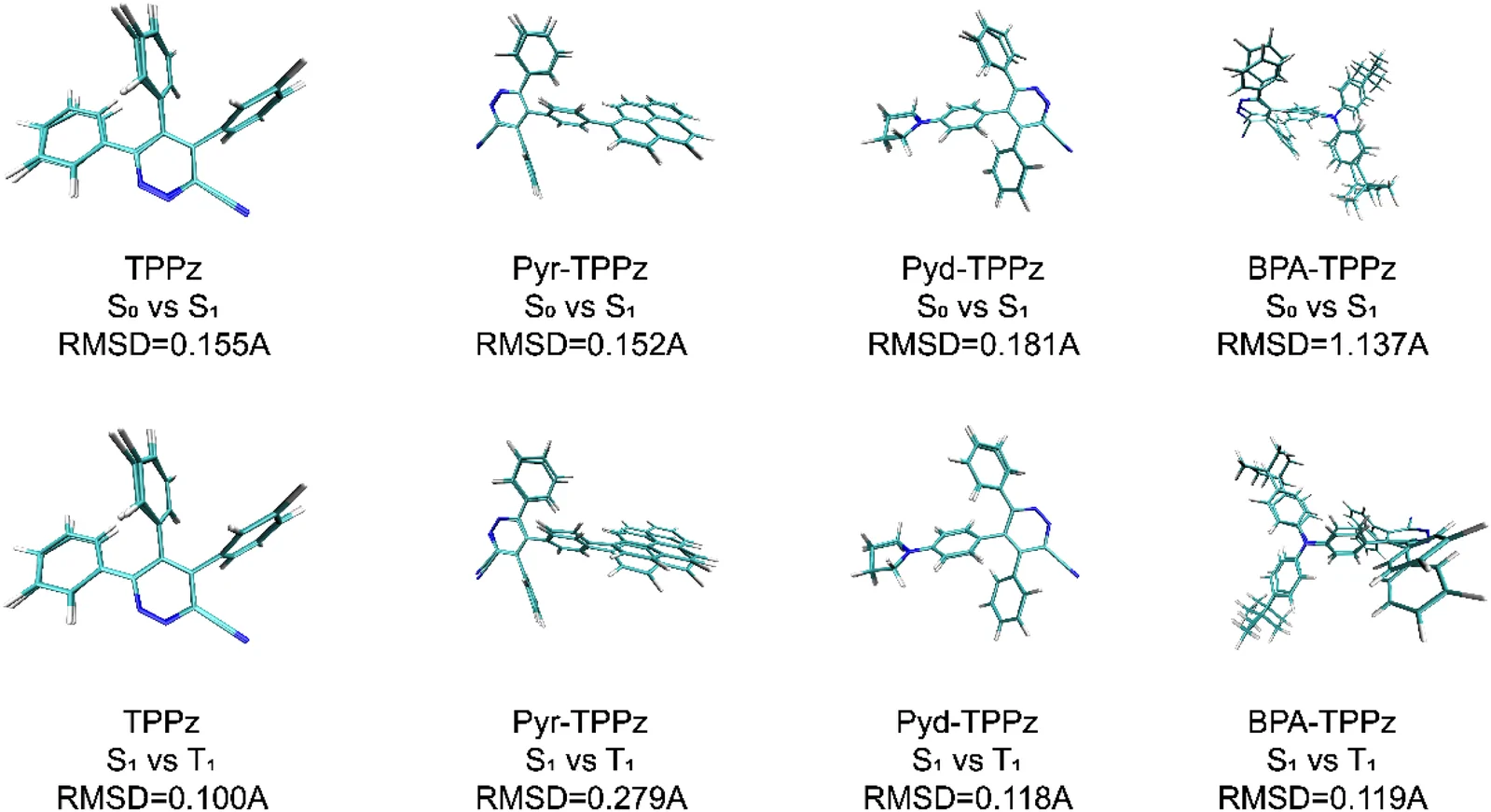
Configurations comparisons of TPPz, Pyr-TPPz, Pyd-TPPz and BPA-TPPz molecule between S_0_, S_1_and T_1_, respectively.

To gain deeper insight into the photophysical properties, we examined the electrostatic potential (ESP) maps of TPPz, Pyr-TPPz, Pyd-TPPz, and BPA-TPPz (Fig. 3). In all molecules, negative charge (blue regions) is significantly concentrated around the TPPz acceptor, with the largest accumulation near the nitrogen atoms. The degree of negative charge on the pyridazine units reflects the electron-withdrawing ability of the TPPz backbone. The frontier molecular orbitals (FMOs) were also analyzed (Fig. 4). The highest occupied molecular orbitals (HOMOs) are mainly distributed over the donor units, while the lowest unoccupied molecular orbitals (LUMOs) reside predominantly on the acceptor. The small overlap between HOMO and LUMO can lead to a reduced singlet–triplet energy gap. For TPPz, the HOMO energy is −6.68 eV, the LUMO energy is −2.18 eV, and the HOMO–LUMO gap is 4.50 eV. For Pyr-TPPz, Pyd-TPPz, and BPA-TPPz, the HOMO energies are −5.48, −5.32, and −5.15 eV, and the LUMO energies are −2.10, −1.83, and −1.95 eV, giving band gaps of 3.38, 3.49, and 3.20 eV, respectively. Steric effects can diminish planarity, which further reduces the HOMO–LUMO overlap and decreases the photoluminescence quantum yield.

**Fig. 3:**
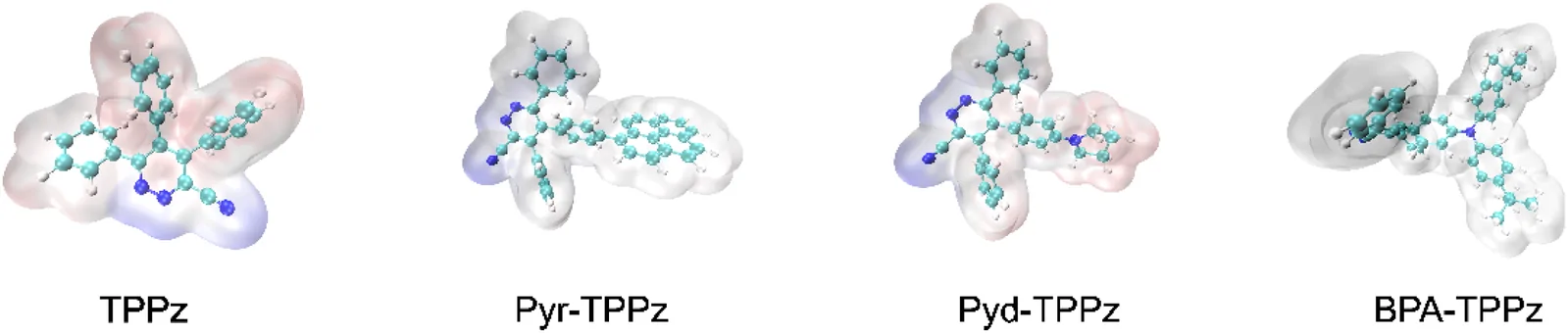
Electrostatic potential distributions of the TPPz, Pyr-TPPz, Pyd-TPPz and BPA-TPPz molecule, respectively.

**Fig. 4:**
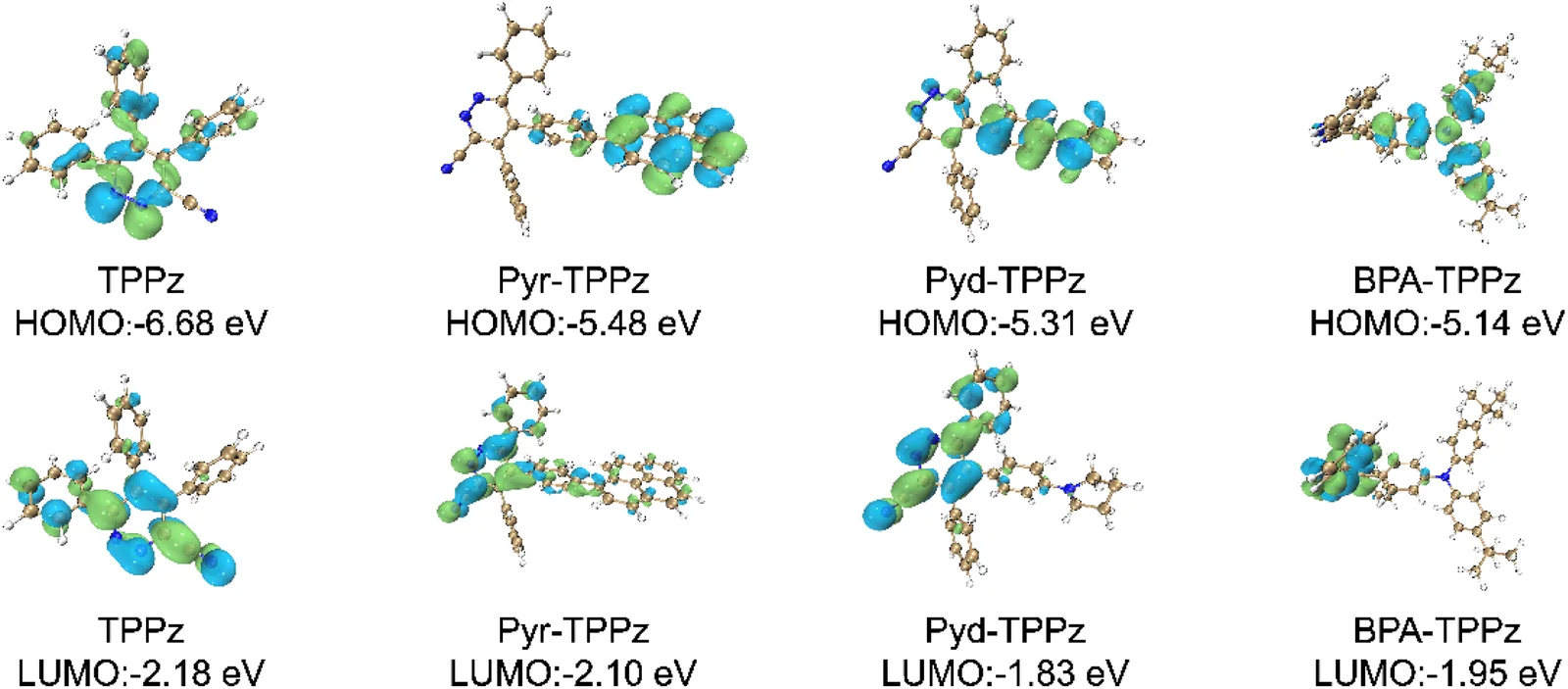
Energy and electron distribution of HOMO and LUMO for TPPz, Pyr-TPPz, Pyd-TPPz and BPA-TPPz molecule in toluene, respectively.

The intersystem crossing (ISC) and reverse intersystem crossing (RISC) processes occur primarily between S_1_ and T_1_. The SOC coefficients between S_1_ and T_*n*_ (*n* = 1–6) were therefore computed using the quadratic response function method as implemented in the Dalton package; the results are summarized in Fig. 5. The SOC constants for TPPz are larger than those of the other molecules, a notable increase that underscores the TADF character of these compounds. Based on the calculated energy landscapes and SOC constants, the ISC rates were obtained theoretically using the TVCF method, and the corresponding data are listed in Table S3. The ISC rate of TPPz is 8.67 × 107 s^−1^, while those of Pyr-TPPz, Pyd-TPPz, and BPA-TPPz are 6.09 × 107, 1.51 × 108, and 2.06 × 108 s^−1^, respectively. The calculated RISC rates (*k*_risc_) at 298 K are 7.89 × 10^2^, 9.91 × 10^−4^, 6.31 × 10^2^, and 8.89 × 10^3^ s^−1^ for TPPz, Pyr-TPPz, Pyd-TPPz, and BPA-TPPz, respectively. Notably, BPA-TPPz exhibits the highest *k*_risc_ among the four molecules, followed by TPPz and Pyd-TPPz, while Pyr-TPPz shows a negligible RISC rate. This trend does not simply follow the SOC magnitude; rather, it reflects the combined influence of SOC, Δ*E*_ST_, and the nature of the T_1_ state. Although TPPz has the largest SOC, its moderate Δ*E*_ST_ and HLCT character lead to a moderate *k*_risc_. BPA-TPPz, despite having moderate SOC, benefits from a nearly pure CT S_1_ state and an HLCT T_1_ state, which together with a small Δ*E*_ST_ and suppressed nonradiative decay, results in the highest *k*_risc_. In contrast, the very low *k*_risc_ of Pyr-TPPz can be attributed to its LE-dominated T_1_ state, which, despite enhanced SOC *via* El-Sayed mixing, is accompanied by a large Δ*E*_ST_ that severely limits the RISC process. Although the S_1_–T_1_ channel is often the primary pathway for RISC, higher-lying triplet states can also participate, particularly when the S_1_–T_1_ gap is large. As shown in Tables S4 and S5, Pyr-TPPz has a large S_1_–T_1_ gap of 0.61 eV and a modest S_1_–T_1_ SOC of 0.286 cm^−1^. However, its S_1_–T_2_ and S_1_–T_3_ gaps are only 0.048 and −0.058 eV, respectively, with SOC values of 0.199 and 0.553 cm^−1^; moreover, S_1_–T_4_ exhibits a much larger SOC of 1.511 cm^−1^ and a moderate gap of −0.169 eV. These S_1_–T_*n*_ (*n* ≥ 2) channels are therefore expected to contribute to the overall RISC process and should not be neglected. For TPPz, the S_1_–T_3_ SOC reaches 6.355 cm^−1^, although the gap is −0.502 eV. For Pyd-TPPz and BPA-TPPz, large SOC values are also found for S_1_–T_2_ (3.525 and 2.52 cm^−1^) and S_1_–T_4_ (5.032 and 5.599 cm^−1^), with gaps of −0.339/−0.876 eV and −0.210/−0.784 eV, respectively. Consequently, the *k*_risc_ values reported in Table S2, which are obtained within the S_1_–T_1_ two-state approximation, should be considered as lower-bound estimates. A full treatment including all S_1_–T_*n*_ channels and their vibrational couplings is desirable for quantitative prediction, while the qualitative trends discussed here remain valid. These results provide a more complete picture of the TADF dynamics and support the conclusion that efficient RISC requires a balance among SOC, Δ*E*_ST_, and CT/LE hybridization. To probe the factors underlying the different behaviors of Pyr-TPPz and BPA-TPPz, we note that these differences are governed not only by their basic electronic and geometric structures but also by their packing modes and intermolecular interactions. The impact of intermolecular interactions on the photophysical properties therefore requires further elucidation.

**Fig. 5:**
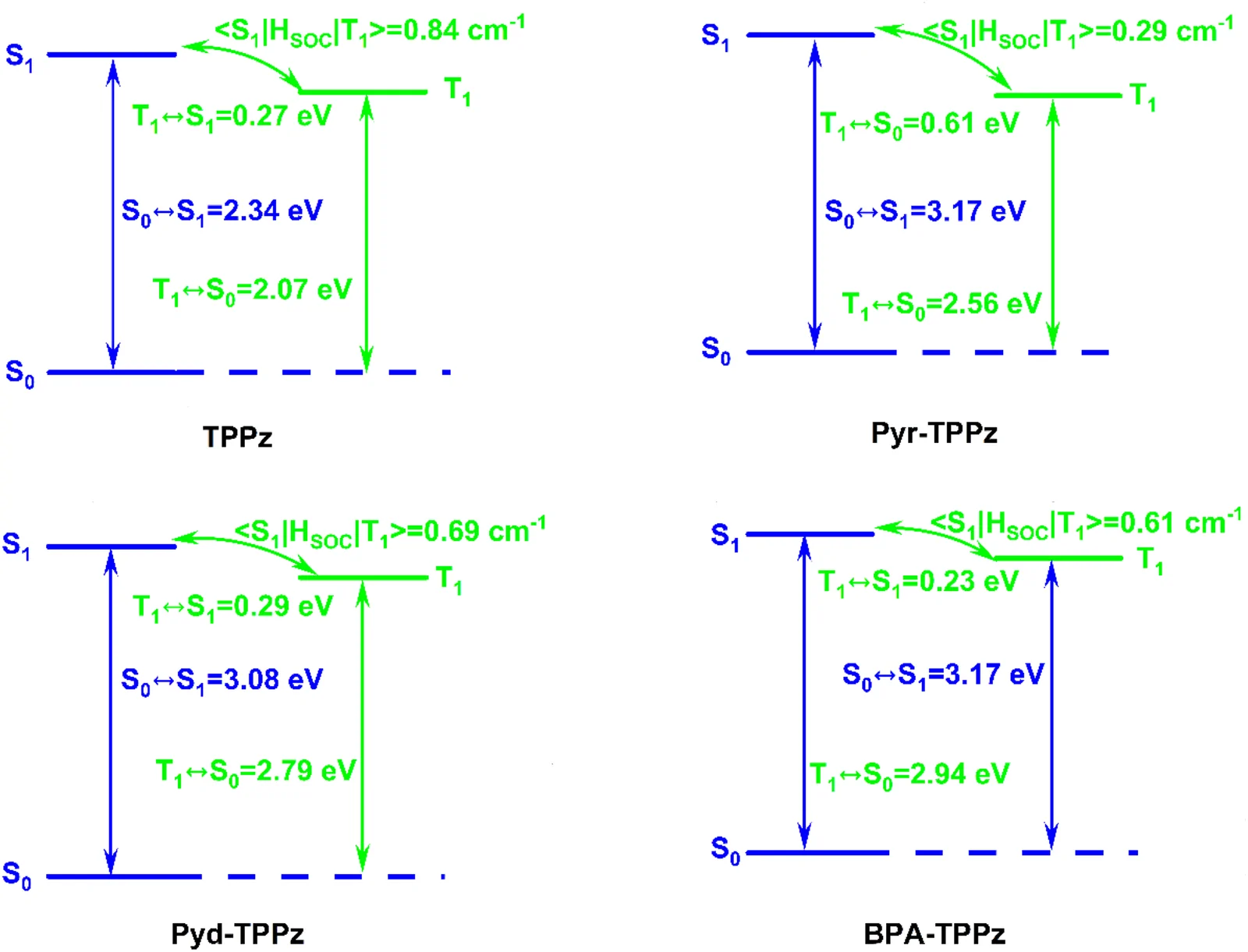
Adiabatic excitation energies of lowest exited states for TPPz, Pyr-TPPz, Pyd-TPPz and BPA-TPPz molecule in toluene, respectively.

To quantitatively characterize the excited-state properties, natural transition orbital (NTO) analyses of S_1_ and T_1_ were performed (Fig. 6 and Table S6). In Fig. 6, the superscripts 1 and 3 denote singlet and triplet states, respectively, and HLCT stands for hybrid local and charge transfer. Thus, ^1^HLCT and ^3^HLCT represent singlet and triplet HLCT states, respectively. Three indices were employed: the locally excited (LE) proportion *S*_r_, the hole–electron centroid distance *D*, and the hole–electron overlap index *t*. According to the LE proportion criterion (*S*_r_ < 0.4 for CT, 0.4 ≤ *S*_r_ ≤ 0.6 for HLCT, *S*_r_ > 0.6 for LE), the S_1_ states can be assigned as follows: TPPz (*S*_r_ = 0.40, borderline HLCT), Pyr-TPPz (*S*_r_ = 0.39, CT), Pyd-TPPz (*S*_r_ = 0.40, borderline HLCT), and BPA-TPPz (*S*_r_ = 0.12, nearly pure CT). The *D* values for the S_1_ state are 1.017, 0.046, 0.756, and 7.644 Å for TPPz, Pyr-TPPz, Pyd-TPPz, and BPA-TPPz, respectively, while the corresponding *t* values are −0.498, −0.313, −0.223, and 6.004 Å. The exceptionally large *D* and small *S*_r_ of BPA-TPPz confirm a pronounced charge-transfer character with a long charge-transfer distance. In contrast, the much smaller *D* values of TPPz, Pyr-TPPz, and Pyd-TPPz indicate limited charge-transfer extent, consistent with their borderline HLCT or CT character. For the T_1_ states, TPPz (*S*_r_ = 0.42, *D* = 0.987 Å, *t* = 0.382 Å) and Pyd-TPPz (*S*_r_ = 0.43, *D* = 0.842 Å, *t* = −0.865 Å) are HLCT, Pyr-TPPz (*S*_r_ = 0.88, *D* = 0.678 Å, *t* = −1.332 Å) exhibits pronounced LE character, and BPA-TPPz (*S*_r_ = 0.44) falls in the HLCT regime. These assignments reveal a more nuanced picture than a simple “twist-grants-CT” paradigm. The rigid pyrenyl (Pyr) donor, despite inducing considerable torsion, yields a CT S_1_ state but a T_1_ state dominated by local excitation on the acceptor. The rigid electron-donating Pyd donor maintains planarity and HLCT character for both states. Only the flexible electron-donating BPA donor simultaneously achieves the highest degree of twisting and the most pronounced CT character in S_1_, while keeping the T_1_ state as HLCT. This differentiation between S_1_ and T_1_ is critical for understanding the intersystem crossing dynamics.

**Fig. 6:**
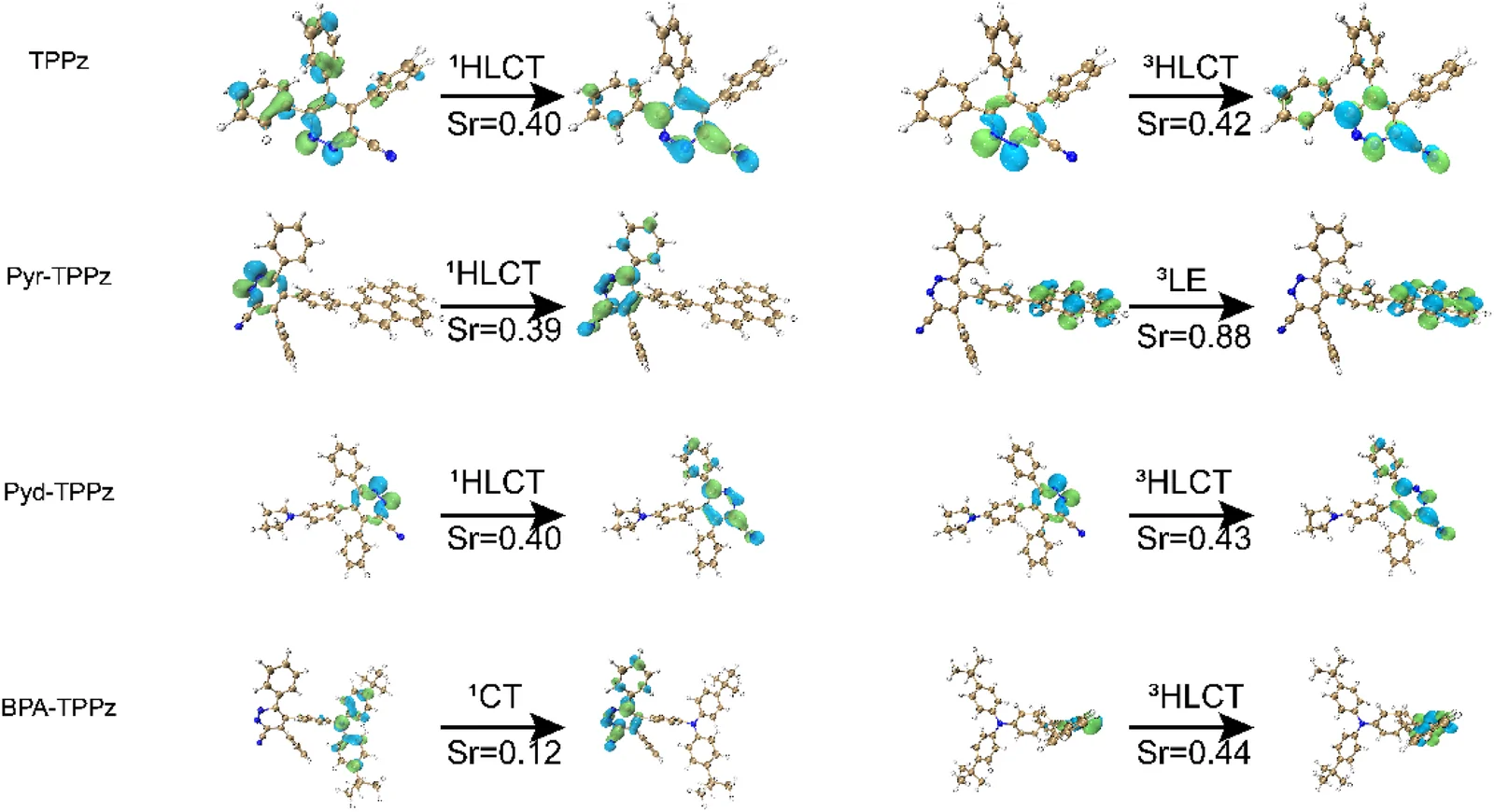
Natural transition orbitals (NTOs) for singlet and triplet states of TPPz, Pyr-TPPz, Pyd-TPPz and BPA-TPPz molecule, respectively.

To uncover the fluorescence quenching mechanism, we calculated the Huang–Rhys (HR) factors and reorganization energies of TPPz, Pyr-TPPz, Pyd-TPPz, and BPA-TPPz, including the Duschinsky effect. Selected contour plots of the absolute values of the Duschinsky rotation matrix are displayed in Fig. 7. The Duschinsky matrix characterizes the reorganization of the potential energy surface upon transitions between different electronic states. Its off-diagonal elements quantify the coupling between vibrational modes in the two states, indicating that a vibrational mode in the ground state may receive contributions from multiple modes in the excited state. Larger off-diagonal elements signify stronger mode mixing and thus more complex nuclear rearrangements. For TPPz, the Duschinsky matrix exhibits significant off-diagonal contributions, whereas for Pyr-TPPz, Pyd-TPPz, and BPA-TPPz the elements are predominantly diagonal, indicating that mode mixing is much more pronounced in TPPz. The involvement of low-frequency motions may render the internal conversion (IC) decay process strongly temperature-dependent. Consequently, all three donor-substituted molecules form effective intramolecular donor–acceptor pairs, as evidenced by the clear spatial separation of HONTO and LUNTO and the dominant CT contribution in S_1_ for Pyr-TPPz and BPA-TPPz. The critical distinction lies in the nature of the T_1_ state: Pyr-TPPz possesses a LE T_1_ state, which enhances spin–orbit coupling *via* El-Sayed allowed mixing between CT singlet and LE triplet states. However, its large S_1_–T_1_ gap makes RISC inefficient within the S_1_–T_1_ two-state approximation. In contrast, BPA-TPPz combines a nearly pure CT S_1_ state with an HLCT T_1_ state, resulting in moderate SOC but benefiting from exceptionally suppressed nonradiative decay.

**Fig. 7:**
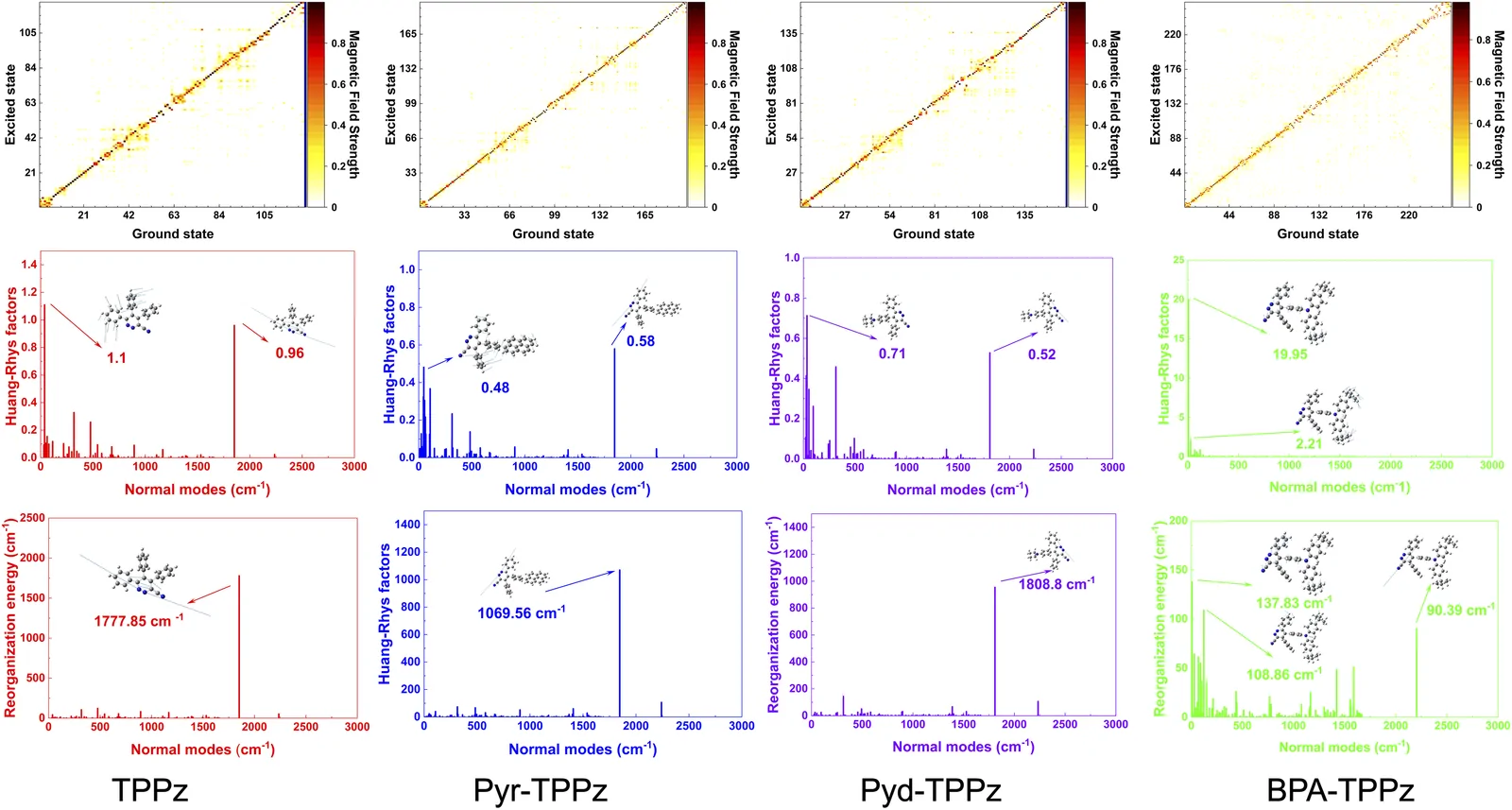
Duschinsky matrix of TPPz, Pyr-TPPz, Pyd-TPPz and BPA-TPPz molecule, respectively. Huang–Rhys factor of TPPz, Pyr-TPPz, Pyd-TPPz and BPA-TPPz molecule, respectively. Reorganization energy of TPPz, Pyr-TPPz, Pyd-TPPz and BPA-TPPz molecule, respectively.

We further analyzed the reorganization energy and HR factors of the four compounds in S_0_ and S_1_. The reorganization energy quantifies the electron–vibrational coupling and the extent of geometric relaxation, while the HR factor characterizes the transfer of vibrational quanta during electronic transitions and is crucial for determining internal conversion rates. Using 
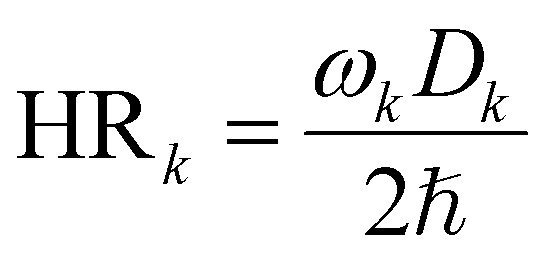
 and 
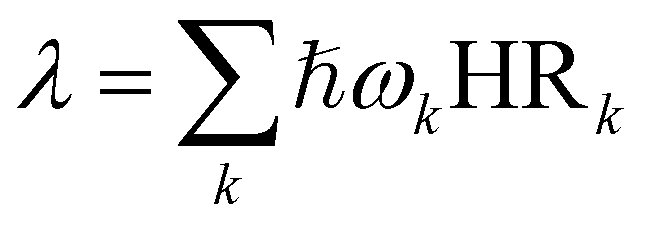
, (where *ω*_*k*_ is the vibrational frequency and *D*_*k*_ is the normal-mode displacement), the HR factors and reorganization energies *λ* were calculated as a function of normal-mode frequencies. To better illustrate the effect of intramolecular interactions, the reorganization energies were projected, and the results are shown in Fig. 7. The data suggest that low-frequency vibrations of the cyanide group cause fluorescence quenching in TPPz. Vibrational modes with HR factors larger than 1.0 are found mainly in the high-frequency region for all compounds. The maximum HR factor of TPPz is 1.1, which is generally much larger than those of Pyr-TPPz, Pyd-TPPz, and BPA-TPPz. At 29.74 and 46.02 cm^−1^, the HR factors for Pyr-TPPz and Pyd-TPPz are 0.48 and 0.71, respectively. These observations highlight the significance of the Duschinsky rotation effect (DRE), which is most pronounced in the low-frequency modes. For clarity, Fig. 7 also presents the normal-mode displacement vectors of the vibrations at 29.74 and 46.02 cm^−1^; both correspond to shear vibrations.

The total reorganization energies of the excited state are 3610.40 cm^−1^ for TPPz, 3826.55 cm^−1^ for Pyr-TPPz, 3465.15 cm^−1^ for Pyd-TPPz, and 2513.42 cm^−1^ for BPA-TPPz. Nonradiative transitions induced by low-frequency vibrational modes below 100 cm^−1^ (∼0.013 eV) can be thermally activated at room temperature (∼0.025 eV) for BPA-TPPz. The reorganization energies of TPPz, Pyr-TPPz, and Pyd-TPPz are very large and concentrated predominantly in the high-frequency region, where fluorescence quenching is temperature-insensitive. In contrast, the introduction of the electron-donating flexible BPA moiety onto the TPPz core yields a markedly smaller and broadly distributed reorganization energy; the total reorganization energy of BPA-TPPz is substantially lower than those of the parent TPPz, Pyr-TPPz (bearing an electron-withdrawing rigid Pyr group), and Pyd-TPPz (bearing an electron-donating rigid Pyd group). This demonstrates that incorporating the flexible BPA moiety effectively reduces the molecular reorganization energy, thereby suppressing the nonradiative transition rate. Consequently, the fluorescence quenching rate of BPA-TPPz is lower than those of the other three molecules.

## Conclusion

In summary, the steric bulk of the donor units dictates molecular planarity: TPPz and Pyd-TPPz exhibit the best overall molecular planarity (smallest MPP/SDP), although their local donor–acceptor dihedral angles remain moderate. NTO analysis shows that the rigid pyrenyl donor in Pyr-TPPz produces a CT S_1_ state but an LE T_1_ state, whereas the flexible electron-donating BPA donor yields a nearly pure CT S_1_ state and an HLCT T_1_ state. BPA-TPPz exhibits the largest excited-state structural distortion yet the lowest reorganization energy (2513 cm^−1^) owing to redistribution of vibrational coupling into low-frequency modes, effectively suppressing nonradiative decay. Within the S_1_–T_1_ two-state approximation, BPA-TPPz shows the highest *k*_RISC, followed by TPPz and Pyd-TPPz, while Pyr-TPPz exhibits a very low *k*_RISC because its large S_1_–T_1_ gap outweighs the SOC enhancement from its LE T_1_ character. These findings establish that efficient TADF emitters require a delicate balance among twisted architecture, tunable CT/LE hybridization in singlet and triplet states to optimize SOC, and flexible donors to minimize reorganization energy.

## Conflicts of interest

The authors declare that they have no known competing financial interests or personal relationships that could have appeared to influence the work reported in this paper.

## Supplementary Material

RA-016-D6RA07215A-s001

## Data Availability

The data supporting this article have been included as part of the supplementary information (SI). Supplementary information is available. See DOI: https://doi.org/10.1039/d6ra07215a.

## References

[cit1] Tang C. W., VanSlyke S. A. (1987). Organic electroluminescent diodes. Appl. Phys. Lett..

[cit2] Uoyama H., Goushi K., Shizu K., Nomura H., Adachi C. (2012). Highly efficient organic light-emitting diodes from delayed fluorescence. Nature.

[cit3] Bunzmann N., Weissenseel S., Kudriashova L., Gruene J., Krugmann B., Grazulevicius J. V., Sperlich A., Dyakonov V. (2020). Optically and electrically excited intermediate electronic states in donor: acceptor based OLEDs. Mater. Horiz..

[cit4] Kothavale S., Lim J., Yeob Lee J. (2022). Rational Design of Cn Substituted Dibenzo[a, c] Phenazine Acceptor for Color Tuning of Thermally Activated Delayed Fluorescent Emitters. Chem. Eng. J..

[cit5] Pei R., Xu Y., Miao J., Peng H., Chen Z., Zhou C., Liu H., Yang C. (2023). A Tetrahedral Bisacridine Donor Enables Fast Radiative Decay in Thermally Activated Delayed Fluorescence Emitter. Angew. Chem., Int. Ed..

[cit6] Pope M., Kallmann H. P., Magnante P. J. (1963). Electroluminescence in organic crystals. J. Phys. Chem. C.

[cit7] Rothberg L. J., Lovinger A. J. (1996). Status of and prospects for organic electroluminescence. J. Mater. Res..

[cit8] Wilson J. S., Dhoot A. S., Seeley A. J. A. B., Khan M. S., Khler A., Friend R. H. (2001). Spin-dependent exciton formation in π-conjugated compounds. Nature.

[cit9] Nehls B. S., Füldner S., Preis E., Farrell T., Scherf U. (2005). Microwave-assisted synthesis of 1,5- and 2,6-linked naphthylene-based ladder polymers. Macromolecules.

[cit10] Adachi C., Baldo M. A., Thompson M. E., Forrest S. R. (2001). Nearly 100% internal phosphorescence efficiency in an organic light-emitting device. J. Appl. Phys..

[cit11] Xiao L., Su S. J., Agata Y., Lan H., Kido J. (2010). Nearly 100% internal quantum efficiency in an organic blue-light electrophosphorescent device using a weak electron transporting material with a wide energy gap. Adv. Mater..

[cit12] Ly K. T., Chen-Cheng R. W., Lin H. W., Shiau Y. J., Liu S. H., Chou P. T., Tsao C. S., Huang Y. C., Chi Y. (2016). Near-infrared organic light-emitting diodes with very high external quantum efficiency and radiance. Nat. Photonics.

[cit13] Penfold T. J., Gindensperger E., Daniel C., Marian C. M. (2018). Spin-vibronic mechanism for intersystem crossing. Chem. Rev..

[cit14] Ahn D. H., Kim S. W., Lee H., Ko I. J., Karthik D., Lee J. Y., Kwon J. H. (2019). Highly efficient blue thermally activated delayed fluorescence emitters based on symmetrical and rigid oxygen-bridged boron acceptors. Nat. Photonics.

[cit15] Lin T.-A., Chatterjee T., Tsai W.-L., Lee W.-K., Meng-Jung W. u., Jiao M., Pan K.-C., Yi C.-L., Chung C.-L., Wong K.-T., Chung-Chih W. u. (2016). Sky-blue organic light emitting diode with 37% external quantum efficiency using thermally activated delayed fluorescence from spiroacridine-triazine hybrid. Adv. Mater..

[cit16] Paras, Ramachandran C. N. (2025). Tuning the electronic and optical properties of phenoxaborin based thermally activated delayed fluorescent materials: A DFT study. J. Fluoresc..

[cit17] Mei Y., Liu D., Li J., Wang J. (2022). Accelerating PLQY and RISC rates in deep-blue TADF materials with the acridin-9(10H)-one acceptor by tuning the peripheral groups on carbazole donors. J. Mater. Chem. C.

[cit18] Shakeel U., Singh J. (2018). Study of processes of reverse intersystem crossing (RISC) and thermally activated delayed fluorescence (TADF) in organic light emitting diodes (OLEDs). Org. Electron..

[cit19] Hua L., Liu Y., Liu B., Zhao Z., Zhang L., Yan S., Ren Z. (2022). Constructing higheffificiency orange-red thermally activated delayed fluorescence emitters by threedimension molecular engineering. Nat. Commun..

[cit20] Tien-Lin W. u., Huang M.-J., Lin C.-C., Huang P.-Y., Chou T.-Y., Chen-Cheng R.-W., Lin H.-W., Liu R.-S., Cheng C.-H. (2018). Diboron compound-based organic light-emitting diodes with high efficiency and reduced efficiency roll-off. Nat. Photonics.

[cit21] Kondo Y., Yoshiura K., Kitera S., Nishi H., Oda S., Gotoh H., Sasada Y., Yanai M., Hatakeyama T. (2019). Narrowband deep-blue organic light-emitting diode featuring an organoboron-based emitter. Nat. Photonics.

[cit22] Yang Y., Zhang Y., Hu X., Liu K., Yang J., Qu Q., Duan Y., Zhang X., Dong L., Liu Y. (2026). Intermolecular Restriction of Intramolecular Vibrations Enables Enhanced Thermally Activated Delayed Fluorescence in the QM/MM ONIOM crystal lattice environment: A QM/MM Study. New J. Chem..

[cit23] Obolda A., Peng Q., He C., Zhang T., Ren J., Ma H., Shuai Z., Li F. (2016). Triplet-polaroninteraction-induced upconversion from triplet to singlet: a possible way to obtain highly efficient OLEDs. Adv. Mater..

[cit24] Etherington M. K., Franchello F., Gibson J., Northey T., Santos J., Ward J. S., Higginbotham H. F., Data P., Kurowska A., Santos P. L. D., Graves D. R., Batsanov A. S., Dias F. B., Bryce M. R., Penfold T. J., Monkman A. P. (2017). Regio- and conformational isomerization critical to design of efficient thermally-activated delayed fluorescence emitters. Nat. Commun..

[cit25] Hojo R., Mayder D. M., Hudson Z. M. (2021). Donor-acceptor materials exhibiting deep blue emission and thermally activated delayed fluorescence with tris(triazolo) triazine. J. Mater. Chem. C.

[cit26] Kothavale S., Chung W. J., Lee J. Y. (2021). High efficiency and long lifetime orange-red thermally activated delayed fluorescent organic light emitting diodes by donor and acceptor engineering. J. Mater. Chem. C.

[cit27] Tang X., Cui L.-S., Li H.-C., Gillett A. J., Auras F., Qu Y. K., Zhong C., Jones S. T. E., Jiang Z.-Q., Friend R. H., Liao L. S. (2020). Highly efficient luminescence from space-confined charge-transfer emitters. Nat. Mater..

[cit28] Kaji H., Suzuki H., Fukushima T., Shizu K., Suzuki K., Kubo S., Komino T., Oiwa H., Suzuki F., Wakamiya A., Murata Y., Adachi C. (2015). Purely organic electroluminescent material realizing 100% conversion from electricity to light. Nat. Commun..

[cit29] Zhang D., Song X., Gillett A. J., Drummond B. H., Jones S. T. E., Li G., He H., Cai M., Credgington D., Duan L. (2020). Efficient and stable deep-blue fluorescent organic lightemitting diodes employing a sensitizer with fast triplet upconversion. Adv. Mater..

[cit30] Jeon S. O., Lee K. H., Kim J. S., Ihn S.-G., Chung Y. S., Kim J. W., Lee H., Kim S., Choi H., Lee J. Y. (2021). High-efficiency, long-lifetime deep-blue organic light-emitting diodes. Nat. Photonics.

[cit31] Yanai T., Tew D. P., Handy N. C. (2004). A new hybrid exchange–correlation functional using the Coulomb-attenuating method (CAM-B3LYP). Chem. Phys. Lett..

[cit32] Weigend F., Ahlrichs R. (2005). Balanced basis sets of split valence, triple zeta valence and quadruple zeta valence quality for H to Rn: Design and assessment of accuracy. Phys. Chem. Chem. Phys..

[cit33] Peach M. J. G., Benfield P., Helgaker T., Tozer D. J. (2008). Excitation energies in density functional theory: An evaluation and a diagnostic test. J. Chem. Phys..

[cit34] Hall D., Sancho-Garcia J.-C., Pershin A., Beljonne D., Zysman-Colman E., Olivier Y. (2023). Benchmarking DFT Functionals for Excited-State Calculations of Donor–Acceptor TADF Emitters: Insights on the Key Parameters Determining Reverse Inter-System Crossing. J. Phys. Chem. A.

[cit35] Hutter J. (2003). Excited state nuclear forces from the Tamm-Dancoff approximation to time-dependent density functional theory within the plane wave basis set framework. J. Chem. Phys..

[cit36] Frisch M. J. , Trucks G. W., Schlegel H. B., Scuseria G. E., Robb M. A., Cheeseman J. R., Scalmani G., Barone V., Petersson G. A., Nakatsuji H., Li X., Caricato M., Marenich A. V., Bloino J., Janesko B. G., Gomperts R., Mennucci B., Hratchian H. P., Ortiz J. V., Izmaylov A. F., Sonnenberg J. L., Williams-Young D., Ding F., Lipparini F., Egidi F., Goings J., Peng B., Petrone A., Henderson T., Ranasinghe D., Zakrzewski V. G., Gao J., Rega N., Zheng G., Liang W., Hada M., Ehara M., Toyota K., Fukuda R., Hasegawa J., Ishida M., Nakajima T., Honda Y., Kitao O., Nakai H., Vreven T., Throssell K., Montgomery Jr J. A., Ogliaro F., Bearpark M. J., Heyd J. J., Brothers E. N., Kudin K. N., Staroverov V. N., Keith T. A., Kobayashi R., Normand J., Raghavachari K., Rendell A. P., Burant J. C., Iyengar S. S., Tomasi J., Cossi M., Millam J. M., Klene M., Adamo C., Cammi R., Ochterski J. W., Martin R. L., Morokuma K., Farkas O., Foresman J. B. and Fox D. J., Gaussian 16, Revision C.02, Gaussian, Inc., Wallingford CT, 2019

[cit37] Lu T. (2024). A comprehensive electron wavefunction analysis toolbox for chemists, Multiwfn. J. Chem. Phys..

[cit38] Lu T., Chen F. (2012). Multiwfn: a multifunctional wavefunction analyzer. J. Comput. Chem..

[cit39] Lu T., Chen Q. (2022). Independent gradient model based on Hirshfeld partition: a new method for visual study of interactions in chemical systems. J. Comput. Chem..

[cit40] Humphrey W., Dalke A., Schulten K. (1996). VMD: visual molecular dynamics. J. Mol. Graph..

[cit41] Shuai Z. (2020). Thermal Vibration Correlation Function Formalism for Molecular Excited State Decay Rates. Chin. J. Chem..

[cit42] Shuai Z., Peng Q. (2014). Excited states structure and processes: Understanding organic light-emitting diodes at the molecular level. Phys. Rep..

[cit43] Shuai Z., Peng Q. (2017). Organic light-emitting diodes: theoretical understanding of highly efficient materials and development of computational methodology. Nat. Sci. Rev..

[cit44] Aidas K., Angeli C., Bak K. L., Bakken V., Bast R., Boman L., Christiansen O., Cimiraglia R., Coriani S., Dahle P., Dalskov E. K., Ekström U., Enevoldsen T., Eriksen J. J., Ettenhuber P., Fernández B., Ferrighi L., Fliegl H., Frediani L., Hald K., Halkier A., Hättig C., Heiberg H., Helgaker T., Hennum A. C., Hettema H., Hjertenæs E., Høst S., Høyvik I.-M., Iozzi M. F., Jansík B., Jensen H. J. A., Jonsson D., Jørgensen P., Kauczor J., Kirpekar S., Kjærgaard T., Klopper W., Knecht S., Kobayashi R., Koch H., Kongsted J., Krapp A., Kristensen K., Ligabue A., Lutnæs O. B., Melo J. I., Mikkelsen K. V., Myhre R. H., Neiss C., Nielsen C. B., Norman P., Olsen J., Olsen J. M. H., Osted A., Packer M. J., Pawlowski F., Pedersen T. B., Provasi P. F., Reine S., Rinkevicius Z., Ruden T. A., Ruud K., Rybkin V. V., Sałek P., Samson C. C. M., Sánchez de Merás A., Saue T., Sauer S. P. A., Schimmelpfennig B., Sneskov K., Steindal A. H., Sylvester-Hvid K. O., Taylor P. R., Teale A. M., Tellgren E. I., Tew D. P., Thorvaldsen A. J., Thøgersen L., Vahtras O., Watson M. A., Wilson D. J. D., Ziolkowski M., Ågren H. (2014). The Dalton quantum chemistry program system. Wiley Interdiscip. Rev.:Comput. Mol. Sci..

[cit45] Angeli K. A. C. , Bak K., Bakken V., Bast R., Boman L., Christiansen O., Cimiraglia R., Coriani S., Cukras J. and Dahle P., Dalton, 2018, vol. 2

