# Supplementary material for: Theoretical insights into the thermally activated delayed fluorescence of pyridazine derivatives: balancing twist and charge-transfer character
Source: RSC Adv. 2026 Oct 8;16(47):61796–805. doi: 10.1039/d6ra07215a (PMC13648323; doi:10.1039/d6ra07215a)
Supplement: Supplementary file 1 [file RA-016-D6RA07215A-s001.pdf]

Supplementary Information (SI) for RSC Advances.
This journal is © The Royal Society of Chemistry

Supporting Information for:

Theoretical Insights into the Thermally Activated Delayed Fluorescence of Pyridazine Derivatives: Balancing Twist and Charge-Transfer Character

Yan Liu[a,b], Shuhua Ma[a], Yuanyuan Yang[a], Chunhua Qu[a], Junyan Zhou[a,d], YuShan Wang[a,d], Yibin Yang[c],* Weiguo Cao[a],*

[a]School of Chinese Materia Medica, Chongqing College of Traditional Chinese Medicine, Chongqing, 402760, China

[b]Chongqing Traditional Chinese Medicine Hospital, Chongqing, 400021, China

[c]Chemical Pollution Control Chongqing Applied Technology Extension Center of Higher Vocational Colleges, Chongqing Industry Polytechnic College, Chongqing, 401120, China

[d]Modern Chinese Medicine Industry College, Chongqing College of Traditional Chinese Medicine, Chongqing, 402760, China

Corresponding Author

*Author to whom correspondence should be addressed.

E-mail: yangyb@cqipu.edu.cn, caoweiguo@cqctcm.edu.cn

Table S1 The Dihedral angle for TPPz, Pyr-TPPz, Pyd-TPPz and BPA-TPPz, respectively.

	S_0	S_1	T_1
TPPz	**51°**	**53.8°**	**51.2°**
Pyr-TPPz	**53.6°**	**59.5°**	**54.0°**
Pyd-TPPz	**56.2°**	**56.5°**	57.3°
BPA-TPPz	**57.6°**	**59.1°**	**56.4°**

Table S2 The MPP and SDP for TPPz, Pyr-TPPz, Pyd-TPPz and BPA-TPPz, respectively.

	MPP/Å			SDP/Å		
	S_0	S_1	T_1	S_0	S_1	T_1
TPPz	0.67	0.63	0.65	2.26	2.23	2.18
Pyr-TPPz	1.13	1.13	1.22	4.90	4.97	5.48
Pyd-TPPz	0.66	0.64	0.63	2.20	2.19	2.02
BPA-TPPz	2.15	1.49	2.18	9.98	6.35	10.24

Table S3 The kr, kic and kisc for TPPz, Pyr-TPPz, Pyd-TPPz and BPA-TPPz, respectively.

	Kr/s^{-1} (S_1→S_0)	$kisc/s^{-1}$ (S_1→T_1)	kic/s^{-1} (S_1→S_0)	$krisc/s^{-1}$ (T_1→S_1)
TPPz	2.37×10^{6}	8.67×10^{7}	5.74×10^{11}	7.89×10^{2}
Pyr-TPPz	9.48×10^{5}	6.09×10^{7}	1.88×10^{11}	9.91×10^{-4}
Pyd-TPPz	4.37×10^{8}	1.51×10^{8}	8.81×10^{10}	6.31×10^{2}
BPA-TPPz	6.37×10^{8}	2.06×10^{8}	1.5×10^{10}	8.89×10^{3}

Note: The *k*risc values were obtained within the S₁–T₁ two-state approximation and should be regarded as lower-bound estimates; contributions from higher-lying triplet states S₁–T□ (n \ge 2*n*≥2) were not included.

Table S4. Spin–orbit coupling (SOC) matrix elements (in cm⁻^{1}) between S1 and Tn (n = 1–6) for TPPz, Pyr-TPPz, Pyd-TPPz, and BPA-TPPz.

	TPPz	Pyr-TPPz	Pyd-TPPz	BPA-TPPz
S1-T1	0.84	0.286	0.688	0.609
S1-T2	1.62	0.199	3.525	2.52
S1-T3	6.355	0.553	2.708	1.825
S1-T4	1.311	1.511	5.032	5.599
S1-T5	0.679	0.211	0.211	0.191
S1-T6	0.587	0.554	0.242	0.229

Table S5. Singlet–triplet energy differences (ΔE_{ST}, eV) between S_1 and T_n (n = 1–6) for TPPz, Pyr-TPPz, Pyd-TPPz, and BPA-TPPz.

	TPPz	Pyr-TPPz	Pyd-TPPz	BPA-TPPz
S_1-T_1	0.27	0.61	0.29	0.23
S_1-T_2	-0.386	0.048	-0.339	-0.21
S_1-T_3	-0.502	-0.058	-0.446	-0.434
S_1-T_4	-1.302	-0.169	-0.876	-0.784
S_1-T_5	-1.5	-0.446	-1.146	-0.912
S_1-T_6	-1.637	-0.485	-1.498	-1.251

Table S6 The NTO for TPPz, Pyr-TPPz, Pyd-TPPz and BPA-TPPz, respectively.

	S_1			T_1		
	S_r	D/Å	t/Å	S_r	D/Å	t/Å
TPPz	0.40	1.017	-0.498	0.42	0.987	-0.382
Pyr-TPPz	0.39	0.046	-0.313	0.88	0.678	−1.332
Pyd-TPPz	0.40	0.756	-0.223	0.43	0.842	-0.865
BPA-TPPz	0.12	7.644	6.004	0.44	7.644	6.004
